# A new autoinhibited kinase conformation reveals a salt-bridge switch in kinase activation

**DOI:** 10.1038/srep28437

**Published:** 2016-06-21

**Authors:** Qiang Wei, Shaoyuan Yang, Dan Li, Xiaoying Zhang, Jimin Zheng, Zongchao Jia

**Affiliations:** 1 College of Chemistry, Beijing Normal University, Beijing, 100875, China; 2 Department of Biomedical and Molecular Sciences, Queen’s University, Kingston, Ontario, K7L 3N6, Canada

## Abstract

In the structure of autoinhibited EphA2 tyrosine kinase reported herein, we have captured the entire activation segment, revealing a previously unknown role of the conserved Arg762 in kinase autoinhibition by interacting with the essential Mg^2+^-chelating Asp757. While it is well known that this Arg residue is involved in an electrostatic interaction with the phospho-residue of the activation loop to stabilize the active conformation, our structure determination revealed a new role for the Arg, acting as a switch between the autoinhibited and activated conformations. Mutation of Arg762 to Ala in EphA2 sensitized Mg^2+^ response, resulting in enhanced kinase catalytic activity and Mg^2+^ cooperativity. Furthermore, mutation of the corresponding Arg/Lys to Ala in PKA and p38MAPK also exhibited similar behavior. This new salt bridge-mediated switch may thus be an important mechanism of activation on a broader scope for kinases which utilize autophosphorylation.

The control of switching on and off of protein kinases is as crucial for their function as their catalytic activity^1^,^2^. Among all the control mechanisms, the key aspect of regulation of most protein kinases is phosphorylation of one or more residues located in the activation segment^3^,^4^,^5^. Upon phosphorylation, the phosphate of the primary phosphorylation site forms multiple interactions both within the activation segment, as well as, other parts of the kinase domain, thereby stabilizing the active conformation. These interactions are highly conserved amongst kinases containing a phosphorylation site within the activation loop, especially the interactions with two positively charged residues^5^.

For example, in the prototypical example of the Ser/Thr kinase cAMP-dependent protein kinase (PKA), the phosphothreonine pThr197 contacts Arg165 which immediately precedes the catalytic base (Asp166), and Lys189 which is only five residues away from the conserved Mg^2+^-chelating aspartate (Asp184)^6^,^7^. Mutational analysis of the catalytic loop Arg (Arg165) corroborates its importance in protein kinases that require activation loop phosphorylation for activation^8^,^9^,^10^. Since the other conserved positively charged residue (Lys189) is in close proximity to the conserved Mg^2+^-chelating Asp184, the interaction between pThr197 and Lys189 is suspected to facilitate the correct conformation of Asp184, as well as, the succeeding residues 185–189^3^,^11^. Indeed the corresponding strand is often disordered or ordered differently in inactive kinases prior to the autophosphorylation of Ser/Thr/Tyr^4^. However, mutation of Lys189 in PKA, or equivalent residue in other kinases, was observed to have no effect on catalytic activity^12^,^13^. Notably, a study of the oncoprotein v-Fps has provided clues to the suspected role of the equivalent positively charged residue Arg1066^10^. In that study, reaction velocity of Arg1066Ala increased sharply at low concentration of Mg^2+^ and the dissociation constant for Mg^2+^ (K_Mg_) of the mutant was much lower than the wild-type, suggesting that Arg1066 is involved with Mg^2+^ affinity. However, until now, the precise role of the conserved positively charged residue, Arg/Lys, and the detailed structural validation of the individual residues involved in catalytic regulation are still not fully understood for PKA, or kinases in general.

In the current study, we have determined the unphosphorylated inactive EphA2 structure at 1.9 Å-resolution, which reveals a previous unknown role of Arg762 (corresponding to Lys189 in PKA) in the autoinhibition state by interacting with the conserved Mg^2+^-chelating Asp757. Furthermore, mutation of the conserved Arg/Lys to Ala in EphA2, as well as, two other Ser/Thr kinases, PKA and p38MAPK, resulted in enhanced catalytic activity and Mg^2+^ cooperativity. These results suggest that the conserved Arg/Lys serves as a novel role of regulating the kinase catalytic activity through modulation of the Mg^2+^ ions binding, which may be an important feature for many protein kinases.

## Methods

### Protein expression and purification

The coding sequence for the human EphA2 intracellular region (residues 583–876) was cloned into MCS1 of pColADuet-1 generating an N-terminal 6× His tag fused to the kinase. The protein was expressed and purified as described previously^14^. The phosphatase, PTEN, was expressed in Sf21 cells and purified using a protocol described previously^14^.

### Crystallization and data collection

Purified EphA2 was treated with a small amount of PTEN for ~2 h at 277 K and then concentrated to ~5 mg/ml for crystallization. The EphA2 was crystallized alone at 293 K by hanging-drop vapour diffusion from 4% Tacsimate pH 7.0, 5% (w/v) PEG3350 and 5% 2-propanol. The data set was collected at the beam line 13B1 of National Synchrotron Radiation Research Center (Hsinchu, Taiwan) and processed with *HKL-3000*^15^.

### Structure determination, refinement and analysis

Molecular replacement was carried out using *Phaser*^16^ using 4P2K as a search model. Model building and refinement were performed using *Coot*^17^ and *phenix.refine*^18^ respectively. The atomic coordinates and structure factors have been deposited in the Protein Data Bank as entry 5EK7. The data collection and refinement statistics are summarized in Table S1.

### Mutagenesis and mutant preparation

The cDNA sequence of the kinase domain of human EphA2 with amino acids 598–876 was cloned into MCS1 of pETDuet-1, eliminating the JMS. Using a PCR-based approach, Tyr772 was mutated to Phe and Arg762 was mutated to Ala. The double mutant derivative, Arg762Ala/Tyr772Phe, was than generated by site-directed mutagenesis using the Tyr772Phe construct as a template. All mutations were confirmed by DNA sequencing. The wild-type and mutant derivatives were expressed purified similar to the wild-type construct.

Full-length murine PKA was cloned into the pFastbacHTA vector to express the protein with an N-terminal 6× His tag. In a similar manner to the EphA2 derivatives, the single mutant Thr197Ala, Lys189Ala and the double mutant Lys189Ala/Thr197Ala derivatives were generated by site-directed mutagenesis. The mutants were confirmed by DNA sequencing. A recombinant baculovirus/Hi5 cell system was used to express the wild-type and mutants of PKA. Recombinant baculovirus was generated using the Bac-to-Bac system (Invitrogen), and Hi5 cells were infected for large-scale protein production. The cells were harvested 36 h post-infection and resuspended in 30 mM MES pH 6.5 with 50 mM KCl supplemented with protease inhibitors and DNase. The protein was then purified as described previously^19^.

The cDNA sequence of murine p38MAPK was cloned into the pET28b vector such that the resultant recombinant clone contains a 6× His tag at the N-terminus. Site-directed mutagenesis was used to generate the Thr180Ala, Arg173Ala and Arg173Ala/Thr180Ala mutants. The wild-type and mutants were confirmed by DNA sequencing and then expressed and purified with reference to previously described protocols^20^.

### Coupled Enzyme Assays

Kinetic analysis of the EphA2, PKA, and p38MAPK mutants was performed using a coupled enzyme assay based on the decrease of absorbance at 340 nm due to NADH oxidation through pyruvate kinase and lactate dehydrogenase. The synthetic peptide used for enzyme kinetics has the sequence DPHTYEDPNQ for EphA2 wild-type and variants^21^, LRRASLG for PKA wild-type and variants^22^, and SMVVPQTPLHTARVLQ for p38MAPK wild-type and variants^23^.

To test the effects of point mutations on kinase Mg^2+^ binding, the 100-μl reaction mixture contained 50 mM HEPES pH 7.5, 1U of lactate dehydrogenase, 1U of pyruvate kinase, 1 mM phosphoenolpyruvate, 0.2 mM NADH, and 2 mM ATP, varying concentrations of Mg^2+^ (0–2 mM or 0–10 mM) and the kinase (0.05–5 μM). Protein concentrations used in the assay were consistent for both the single and double mutant derivatives of each kinase with Tyr-to-Phe/Thr-to-Ala mutation; for EphA2 variants, the protein concentration used was 0.2 μM, while the PKA variants were used at 0.03 μM, and the p38MAPK variants at 3 μM. The reactions were initiated by the addition of 1 mM of the relevant peptide substrate. All experiments were performed in triplicate at room temperature. Reaction velocities were determined by dividing the increase in ADP concentration in the mixture (μM) by the reaction time (min). The Hill coefficient was calculated using the sigmoidal Hill equation as follows:


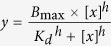


where: *y* = measured velocity, *B*_*max*_ = maximal velocity at saturating magnesium binding, *x* = tested magnesium concentration, *K*_*d*_ = the calculated magnesium concentration that elicits 50% of maximal velocity and *h* = Hill coefficient.

The steady-state kinetic parameters *K*_*peptide*_ and *K*_*ATP*_ for the variants were determined by plotting the reaction velocity versus either the total substrate concentration at fixed ATP concentrations or the total ATP concentration at fixed substrate peptide concentrations. The *K*_*peptide*_ was tested using fixed concentrations of ATP (2 mM) and MgCl_2_ (10 mM for EphA2 and PKA variants; 2 mM for p38MAPK variants) with varying concentrations of the substrate peptide (0.1–3 mM). The *K*_*ATP*_ was tested using fixed concentrations of substrate (2 mM) and MgCl_2_ (10 mM for EphA2 and PKA variants; 2 mM for p38MAPK variants) with varying concentrations of ATP (0.1–5 mM). Protein concentrations of 0.1 μM to 2 μM were used in the assay. The *K*_*peptide*_ and *K*_*ATP*_ values were calculated using the Michaelis-Menten equation using Sigmaplot 9.0.

## Results and Discussion

**Figure 1:**
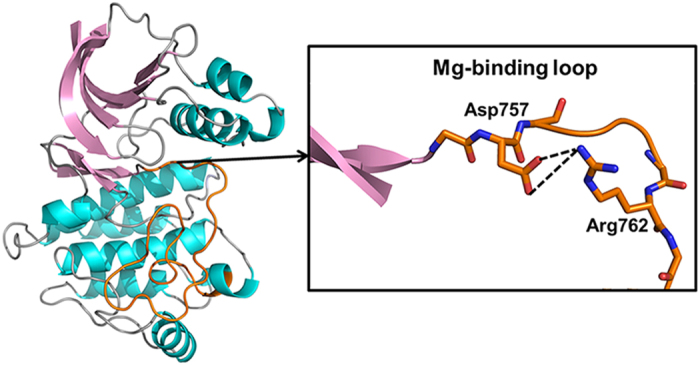
Ribbon representation of the structure of autoinhibited EphA2. The N-terminal lobe (N-lobe) contains five β strands (1 through 5; colored pink), the JMS and a conserved αC-helix. The C-lobe is mostly helical (colored cyan). The loops are colored grey while the activation segment is colored orange. The “Mg^2+^-binding loop” in the activation segment is highlighted in the boxed insert. The salt-bridge interactions between Asp757 and Arg762 in the activation segment are shown as black dashed lines.

A cytoplasmic fragment (residues 583 to 876) of EphA2 consisting of the entire catalytic domain and the C-terminal half of the juxtamembrane segment (JMS) was expressed in *E. coli*. Since our previous research established the enzyme-substrate reciprocal relationship between EphA2 and PTEN^14^, we treated EphA2 using PTEN phosphatase to obtain an unphosphorylated autoinhibited EphA2 structure. The data collection and refinement statistics are summarized in Table S1. The EphA2 structure shows an overall bi-lobed structure typical of kinases (Fig. 1). Compared with our previously reported autoinhibited EphA2 structure (PDB 4PDO, crystallized under different conditions with different space group) in which the activation segment was not observed due to poor electron density, the present EphA2 structure exhibits a complete activation segment (Fig. S1). This structure provides a first glimpse of previously unknown features that enable an autoinhibited conformation, one of which is the Arg762-Asp757 interaction (Fig. 1 and Fig. S2). Even though a few crystal contacts are observed for this newly observed conformation of the activation segment, the fact that there are two independent molecules in the asymmetric unit may, to some extent, alleviate the concerns. Because it is difficult to ascertain if the contacts influence the observed conformation of the activation segment just on the basis of the structure, we have subsequently prepared relevant mutants and carried out kinetic experiments to test the crystallographic findings and the results corroborate the autoinhibitory role of Arg762 (see below). We thus believe that the crystal contacts do not appear to play a significant role. The N-terminal portion of the activation segment, termed “magnesium-binding loop”, contains the conserved DFG motif (residues 757–759) (Fig. S3), in which the aspartate residue (Asp757) is crucial for Mg^2+^-coordination^5^. For most kinases, Mg^2+^ ions are required to catalyze phosphoryl transfer and increase the enzyme’s affinity for ATP, and, as such, proper coordination of Mg^2+^ ions is of critical importance^21^,^24^,^25^. Hence, the observation of strong interaction between the Mg^2+^-chelating Asp757 and the conserved positively charged residue, Arg762, is rather intriguing.

**Figure 2:**
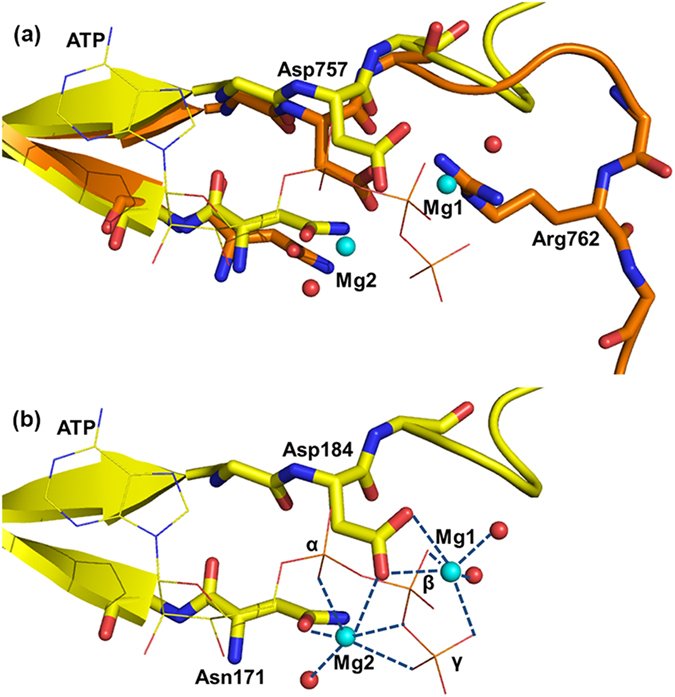
The novel interaction between Asp757 and Arg762 in the activation segment of the autoinhibited EphA2 structure in comparison with the PKA structure activation segment. (**a**) Alignment of the N-terminal part of the activation segment from EphA2 and PKA. The backbone of the activation segment of EphA2 is colored in orange, and PKA is shown in yellow. ATP from the PKA structure is depicted as lines and colored by element, the coordinating water molecules are colored red, and Mg^2+^ ions are colored cyan. (**b**) View of Mg^2+^ coordination in PKA. Metal coordination is shown as blue dashed lines.

To estimate the effect of the newly observed interaction, the activation segment of the inactive EphA2 structure was overlaid with the activation segment of PKA together with Mg^2+^ ions and ATP (Fig. 2a). There are two Mg^2+^ binding sites in PKA (Fig. 2b). One Mg^2+^ ion (termed Mg1) is coordinated by Asp184 (Asp757 in EphA2), the β- and γ-phosphates of ATP, and two water molecules. The other Mg^2+^ ion (termed Mg2) is coordinated by one oxygen atom of Asp184 (Asp757 in EphA2), Asn171 (Asn744 in EphA2), the α- and γ-phosphates of ATP, and one water molecule^26^. Thus, the negatively charged Asp of the DFG motif participates in the coordination of both Mg1 and Mg2. However, the double salt-bridge interaction between Asp757 and Arg762 would draw electrons away from the oxygen atom of the carboxylate group to weaken or even prevent Mg^2+^ binding by Asp757 in the inactive state, thus severely compromising its ability to coordinate the Mg^2+^ ions. Consequentially, the optimal positioning and/or binding of Mg^2+^ ions would be impaired, leading to autoinhibition and impairing the kinase’s catalytic properties. Furthermore, as shown in Fig. 2a, the orientation of Arg762 in the inactive EphA2 structure may result in steric clash with Mg1. The repulsion between the positively charged Arg762 and Mg^2+^ ions would also impede the optimal coordination of Mg1 and Mg2. Taken together, our result marks the first observation of a novel autoinhibition mechanism featuring the interference of Mg^2+^ binding.

Previous work on autoinhibition mechanisms involving the N-terminal anchor of the activation segment almost always exhibit rearrangement of the N-terminal residues, typically leading to a “DFG-out” conformation of the Mg^2+^ binding loop^4^. Thus, in these autoinhibited structures, although complete activation segment was also visible in the electron density maps, the equivalent salt-bridge could not be observed because of the disrupted N-trerminal configuration which results in a different position of the conserved Arg/Lys equivalent to Arg762. In most cases, large structural rearrangement of the N-terminal anchor is resulted from distortion of the activation loop, such as the autoinhibited insulin receptor tyrosine kinase (IRK)^27^ and muscle-specific kinase (MuSK)^28^. In some kinases, however, even subtle positional changes in the activation loop can be amplified into large structural changes of the N-terminal anchor. For instance, in the inactive Bruton’s tyrosine kinase (Btk) structure, the repositioning of the phosphorylatable Tyr causes a distortion in the Mg^2+^ binding loop, leading to the disruption of the active site residues^29^. In contrast, in the inactive EphA2 structure, Arg762 interacts directly with the critical Mg^2+^-binding residue, Asp757, without the concomitant large structural changes of the N-terminal residues of the activation segment. As shown in Fig. 2, the ATP binding site is not perturbed and Asp757 remains in the same place; even the DFG phenylalanine maintains its “DFG-in” conformation. The proximity between Asp757 and Arg762, which are only five residues apart, may facilitate the interaction without the need for a large structural rearrangement.

Moreover, the C-terminal section of the activation segment also serves an autoinhibitory role by interfering with substrate peptide binding. Alignment of the ternary IRK3P and the inactive EphA2 structure shows that the residues immediately preceding the PTK-invariant Pro780 (corresponding to Pro1172 in IRK3P) of EphA2 are in much different positions than their counterparts in IRK3P (Fig. S4a), which are critical residues required to accommodate the substrate peptide. As shown in Fig. S4b, in the ternary IRK3P structure, which consists of the phosphorylated, activated form of the insulin receptor Tyr kinase, a peptide substrate and an ATP analog, the C-terminal residues of the activation segment form a short β-strand with the observed residues of the substrate by pairing in an anti-parallel manner^30^. Instead, the corresponding residues of EphA2 participate in a unique set of intramolecular contacts (Fig. S4c) and are unfavorably positioned for substrate binding, leading to severe spatial clashes between the superimposed substrate and the activation segment of EphA2 (Fig. S4d). Thus, the residues at the C-terminal end of the activation segment are positioned in a way which hinders binding of a substrate peptide rather than accommodates it, which is similar to the autoinhibition mechanism revealed by the FGFR1K structure^31^.

**Figure 3:**
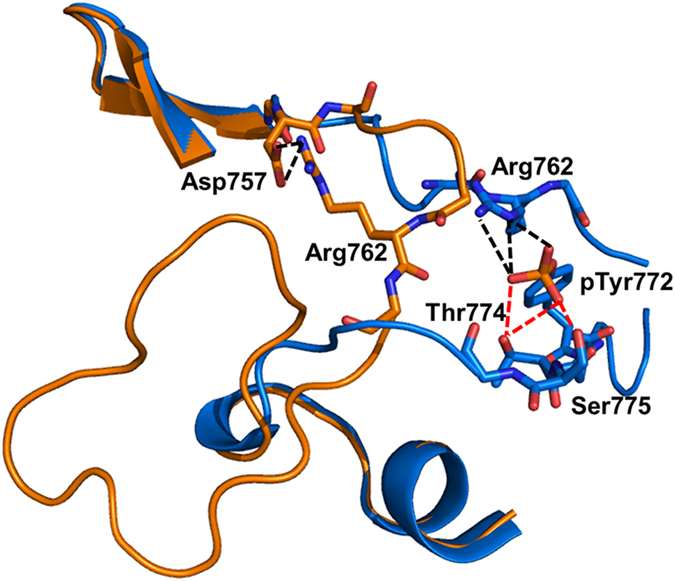
Alignment of the activation segment of the autoinhibited and active EphA2 (PDB 4TRL). The backbone of the active EphA2 is colored marine. The salt-bridge interactions between the pTyr772 and Arg762 are shown as black dashed lines and the selected hydrogen-bonding interactions are shown as red dashed lines. The backbone of the autoinhibited EphA2 is colored as in Fig. 2 and the salt-bridge interactions between Asp757 and Arg762 are shown as black dashed lines.

In order to better understand the mechanism by which Tyr phosphorylation in the activation segment of EphA2 causes a switch from the inactive to the active state, we aligned the autoinhibited structure with our previously solved active EphA2 structure^14^ (Fig. 3). Upon phosphorylation of Tyr772, the phosphate moiety interacts with residues in the activation segment, such as Arg762, Thr774 and Ser775. Interactions of pTyr772 with Thr774 and Ser775 may help the C-terminal end of the activation segment adopt a correct conformation for substrate binding. The double salt-bridge interaction between Arg762 and Asp757 is completely broken as Arg762 is pulled away from the Asp residue by the new phosphate group and replaced in the active state by electrostatic interaction of the former with pTyr772. The shift of Arg762, as it is competed away from the aspartate by the new phosphate moiety, serves to extend the conformation of the N-terminal half of the activation segment and the formation of accessible Mg^2+^ binding sites (Fig. S5).

Thus, our observation of the interaction between Arg762 and Asp757 reveals a novel regulatory role of the conserved positively charged Arg/Lys residue. In a typical “relationship triangle”, the breakup of the Arg-Asp interaction is caused by the formation of new electrostatic interaction between the phosphotyrosine and Arg762, a novel feature in kinase activation. Given the highly conserved nature of the Asp and the Arg/Lys residues and the close proximity between the two residues, we expected that this newly discovered mechanism would exist in other eukaryotic protein kinases (EPKs).

**Figure 4:**
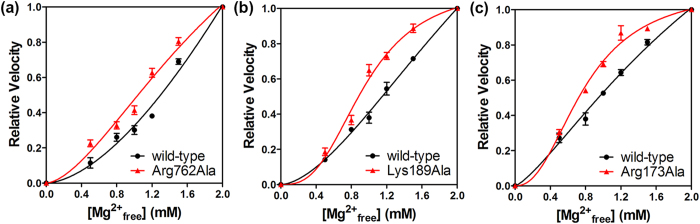
Plots of relative velocity versus different Mg^2+^ concentration for the wild-type and variant of EphA2, PKA and p38MAPK. Each experiment was performed in triplicate (the error bar represents standard deviation). (**a**) The velocities of the wild-type (black) and Arg762Ala variant (red) of EphA2 are normalized to the velocities attained at 2 mM of Mg^2+^. (**b**) The velocities of the wild-type (black) and Lys189Ala variant (red) of PKA are normalized to the velocities attained at 2 mM of Mg^2+^. (**c**) The velocities of the wild-type (black) and Arg173Ala variant (red) of p38MAPK are normalized to the velocities attained at 2 mM of Mg^2+^.

In order to test our crystallographic finding of the autoinhibitory role of Arg762, we generated site-directed mutations in the EphA2 kinase domain. Since the interaction of Arg762 with Asp757 would serve to interfere with Mg^2+^ binding, Arg762 was mutated to alanine to abolish the salt bridge. The effect of Arg-to-Ala mutation was examined by comparing the relative velocities of the Arg762Ala mutant and wild-type (Fig. 4a and Table S2). At concentrations greater than 0.5 mM Mg^2+^, it was found that the Arg762Ala mutant showed a greater relative velocity compared to the wild-type. From this, it can be inferred that the affinity of Mg^2+^ ions is enhanced by the replacement of Arg762 with Ala and confirms that Arg762 is not conducive to Mg^2+^ binding.

**Figure 5:**
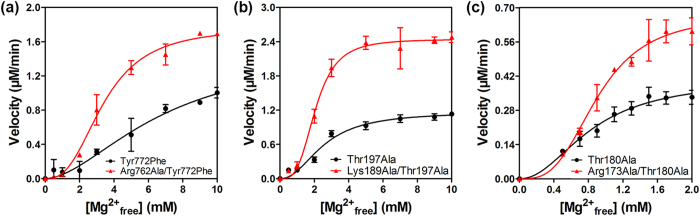
Effects of free Mg^2+^ on the activation of EphA2, PKA, and p38MAPK mutants. Each experiment was performed in triplicate (the error bar represents standard deviation). (**a**) Reaction velocity of the EphA2 mutants Tyr772Phe (black) and Arg762Ala/Tyr772Phe (red) are measured at increasing Mg^2+^ concentration in the range of 0–10 mM. (**b**) Reaction velocity of the PKA mutants Thr197Ala (black) and Lys189Ala/Thr197Ala (red) are measured in a similar manner to EphA2. (**c**) Reaction velocity of the p38MAPK mutants Thr180Ala (black) and Arg173Ala/Thr180Ala (red) are measured at Mg^2+^ concentrations in the range of 0–2 mM.

Further, since the Arg-Asp salt-bridge would be broken up by the formation of salt-bridge interactions between the pTyr772 and Arg762 upon phosphorylation in wild-type, a Tyr772Phe mutant was generated to avoid the effect of phosphorylation, thus, acting as a control instead of the wild-type. To specifically investigate the function of Arg762, the Tyr772-to-Phe mutation was also introduced into the Arg762Ala mutant. Consequently, both single (Tyr772Phe) and double mutant (Arg762Ala/Tyr772Phe) derivatives were generated. The effect of arginine-to-alanine mutation was examined by measuring kinase activity (Fig. 5 and Table S3). The reaction velocity was measured at increasing Mg^2+^ concentrations for the two variants at the same protein concentration, in conditions of fixed peptide and ATP concentrations. With the phosphorylation site mutated, this group of variants demonstrated more apparent difference in a broader range of Mg^2+^ concentration. As illustrated in Fig. 5a, the reaction velocity of the double mutant is ~2-fold higher than that of the single mutant derivative, indicating higher kinase activity upon removal of the Arg-Asp salt-bridge by the Arg-to-Ala mutation. More importantly, the Hill coefficient for the double mutant was larger than for the single mutant (Arg762Ala/Tyr772Phe *n*_*H*_ = 2.69 ± 0.35, Tyr772Phe *n*_*H*_ = 1.78 ± 0.45, *n* = 3; *p* < 0.05, Student’s *t* test (independent, two tailed)), while as expected either the affinity for substrate or the *K*_*m*_ for ATP was not significantly affected by the mutation of Arg762 to Ala (Table S4) since Arg762 is not directly involved in cofactor and substrate binding. The Hill coefficient is thought to reflect the extent of cooperativity among multiple ligand binding sites^32^, and may suggest that Arg762 affects kinase activity by affecting Mg^2+^ coordination as its mutation to Ala caused enhanced Mg^2+^ cooperativity. These results are consistent with the crystallographic finding that the position of Arg762 would interfere with the binding of Mg^2+^ ions, resulting in Mg^2+^-mediated effect which contributes to the inactive state. Moreover, the autoinhibition mechanism revealed in this work would provide a rationalization for another Tyr kinase, v-Fps mentioned above, in which the equivalent mutant Arg1066Ala was found to bind Mg^2+^ with an affinity which is 23-fold stronger than the wild-type^10^.

In order to determine if the Arg autoinhibition mechanism discovered in EphA2 Tyr kinase is widely applicable to other kinases, particularly Ser/Thr kinases regulated by autophosphorylation, we expressed and purified two Ser/Thr kinases from different EPKs subfamilies, including PKA from the AGC subfamily and the p38MAPK from the CMGC subfamily. Similar to EphA2, to test the autoinhibitory role of the conserved Arg/Lys, the corresponding residue was mutated to Ala. Consequently, the Lys189Ala mutant of PKA and Arg173Ala mutant of p38MAPK were generated. For either kinase, the variant displays greater relative velocity compared to the wild-type (Fig. 4b,c), indicating enhanced Mg^2+^ response by the replacement of Arg/Lys with Ala.

Further, the Thr primary phosphorylation site was mutated to Ala to remove the effect of phosphorylation. As such, both single Thr197Ala and double Lys189Ala/Thr197Ala mutant derivatives of PKA, as well as, single Thr180Ala and double Arg173Ala/Thr180Ala mutant derivatives of p38MAPK were generated. The effects of mutagenesis on the activity profiles of these two kinases are consistent with those seen with EphA2 (Fig. 5b,c). For either kinase, with the phosphorylation site mutated, the double mutant exhibits higher kinase activity and a larger Hill coefficient compared with the respective single mutant derivative (PKA: Lys189Ala/Thr197Ala *n*_*H*_ = 3.37 ± 0.42, Thr197Ala *n*_*H*_ = 2.19 ± 0.54, *n* = 3, *p* < 0.05, Student’s *t* test (independent, two tailed); p38MAPK: Arg173Ala/Thr180Ala *n*_*H*_ = 3.13 ± 0.31, Thr180Ala *n*_*H*_ = 2.08 ± 0.45, *n* = 3, *p* < 0.05, Student’s *t* test (independent, two tailed)), while displaying only slightly different *K*_*m*_ for substrate peptide and ATP (Table S4). The results confirm that the conserved Lys/Arg serves a unique function in autoinhibition by destabilizing Mg^2+^ binding. Although ~2-fold activity difference beween the double and single mutants seems very small, it should be stressed that this increased kinase activity is achieved on the basis of Tyr-to-Phe or Thr-to-Ala mutant. In the context of the unphosphorylatable mutant which possesses much diminished activity and perhaps reduced sensitivity, further change may be more difficult to achieve. Thus, the main point here is not how much Arg/Lys-to-Ala mutation could improve kinase activity, but the fact that it can result in enhanced activity and Mg^2+^ cooperativity. Whereas compromising protein’s properties through mutations is widely observed, the opposite effect (enhancement) is much less common and often more revealing in understanding protein’s function. Given the consistency of these functional observations, the findings stemming from our EphA2 inactive structure may present an additional mechanism utilized by many protein kinases.

Although the importance of Mg^2+^ ions is widely recognized in phosphoryl transfer, the modulation of the metal coordination is poorly understood for kinases in general. There is a lot of controversy about the binding of the two Mg^2+^ ions. Mg1 was thought to bind first while Mg2 was identified as the secondary and inhibitory ion partially based on early low-resolution structures of PKA^33^. However, the more recent, higher-resolution structure of PKA obtained under low Mg^2+^ concentration showed density for only Mg2^26^. Further, subsequent structural characterizations of PKA and CDK2 suggested that Mg2 is likely the first and last remaining Mg^2+^ ion in the active site with Mg1 being expelled following phosphoryl transfer^34^,^35^. Despite these contrasting theories, both hypotheses imply that Mg^2+^ coordination is a highly regulated process, and that Mg1 and Mg2 are neither bound nor expelled simultaneously, a notion which is supported by the smaller Hill coefficients of the single mutant derivatives compared to the double mutants presented here. Moreover, the inactive EphA2 structure shows strong interaction between the Mg^2+^-chelating Asp and the conserved Arg, which affects the Mg^2+^ coordination, especially Mg1. Therefore, our results corroborate the latter theory that Mg1 binding is less favored than Mg2, by providing an underlying structural basis for both the observation and understanding the modulation of Mg^2+^ binding.

## Conclusion

The observations presented here provide structural evidence for the regulation of kinase activity through modulation of Mg^2+^ binding via a salt-bridge switch between Arg/Lys with the Mg^2+^-chelating Asp and Arg/Lys with the activating phosphorylation site. The autoinhibited structure reveals a mechanism of autoinhibition through interference with the binding of Mg^2+^, as well as, substrate, which is relieved by autophosphorylation of the activation segment. The investigation of the mutation of Arg/Lys to Ala in both tyrosine kinase (EphA2) and serine/threonine kinases (PKA and p38MAPK) exhibits enhanced kinase catalytic activity and Mg^2+^ cooperativity. Thus, the conserved Arg/Lys residue can be considered to be the “gatekeeper” by governing Mg^2+^ binding; the modulation of Mg^2+^ binding through this conserved Arg/Lys gatekeeper may be an important mechanism for most EPKs regulated by autophosphorylation in the activation loop.

## Additional Information

**How to cite this article**: Wei, Q. *et al*. A new autoinhibited kinase conformation reveals a salt-bridge switch in kinase activation. *Sci. Rep.* **6**, 28437; doi: 10.1038/srep28437 (2016).

## Supplementary Material

Supplementary Information
