# Supplementary material for: A new autoinhibited kinase conformation reveals a salt-bridge switch in kinase activation
Source: Sci Rep. 2016 Jun 21;6:28437. doi: 10.1038/srep28437 (PMC4914941; doi:10.1038/srep28437)
Supplement: Supplementary Information [file srep28437-s1.pdf]

Supporting information

A new autoinhibited kinase conformation reveals a salt-bridge switch in kinase activation

Qiang Wei, Shaoyuan Yang, Dan Li, Xiaoying Zhang, Jimin Zheng*, and Zongchao Jia*

*co-corresponding authors (jimin_z@bnu.edu.cn; jia@queensu.ca)

Table S1 Crystallographic statistics

**Crystals**	Autoinhibited EphA2 JMS-KD (583-876)
**Data Collection**	
Space group	$P2_1$
Unit-cell dimensions (length Å; angle °)	a=63.5, b=75.2, c=80.5; α=γ=90, β=112.7
Resolution range(Å)	30.0-1.9 (1.97-1.90) [b]
R_{sym}(%) [a]	6.0 (30.4) [b]
Unique reflections	54658 (5471) [b]
Completeness (%)	99.7 (99.7) [b]
<I/σ(I)>	20.9 (4.1) [b]
Multiplicity	3.6 (3.5) [b]
**Refinement**	
Resolution (Å)	26.8-1.9 (1.93-1.90) [b]
R_{work}/ R_{free} (%)	17.1 (19.4) [b]/20.5 (23.9) [b]
RMSD bond length (Å)/angle (°)	0.009/1.146
**No. of molecules per asymmetric unit**	2
**No. of atoms**	
Protein	4568
Water	832
**Average B factors (Å^2)**	
Protein	20.9
Water	32.1
**Ramachandran plot, residues in (%)**	
Most favored regions	97.9
Additional allowed regions	2.1
disallowed	0
PDB ID	5EK7

[a] $R_{sym}=\Sigma(|I - < I >|)/\Sigma(I)$, where I is the observed intensity and < I > is the average intensity of all measured observations equivalent to reflection I.
[b] Numbers in parenthesis represent values in the highest resolution shell.

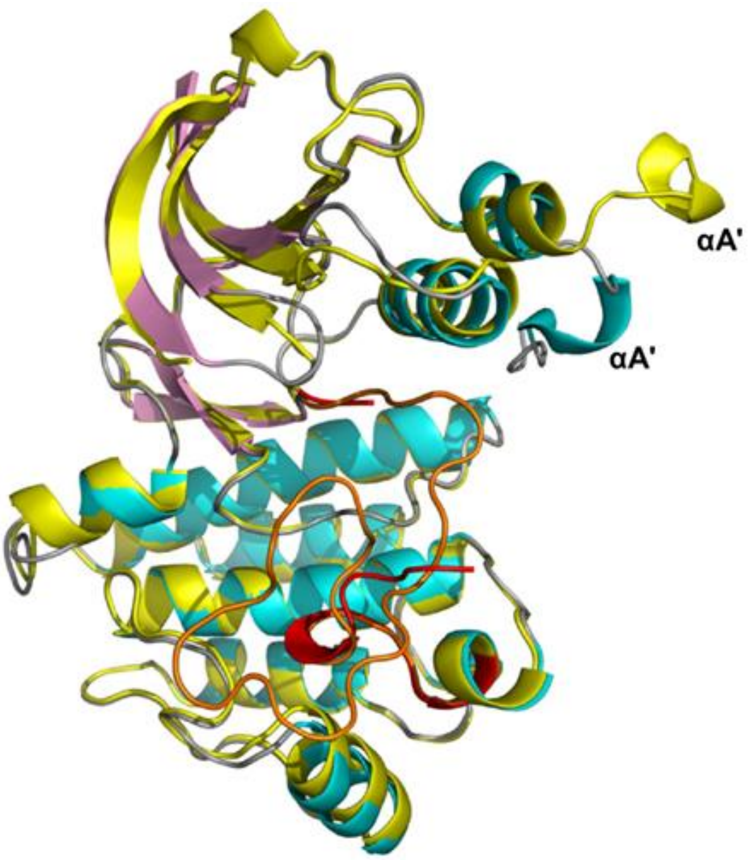

Figure S1. Superposition of the autoinhibited structure of EphA2 and the previously solved inactive structure (PDB 4PDO). The backbone of the new EphA2 structure is colored as in Figure 1. The backbone of the previous structure is depicted in yellow, and the remaining activation segment in red. The JMS of the newly solved inactive EphA2 structure, especially the N-terminus, adopts a position distinct from the previous structure but similar with the autoinhibited EphB2, by extending along the cleft region between the N- and C-terminal lobes.

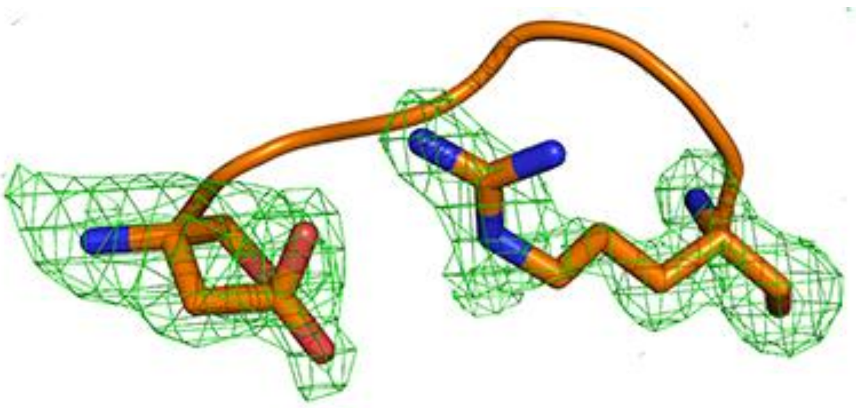

Figure S2. The omit *Fo-Fc* electron density map of the critical Mg^{2+}-binding residue Asp757 and Arg762 at 3 σ level in the activation segment of the autoinhibited EphA2 structure.

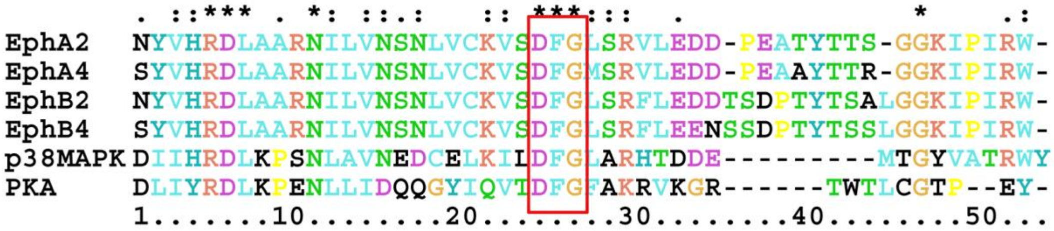

Figure S3. Alignment of the activation segment of four representative Eph receptors (EphA2, EphA4，EphB2 and EphB4) and the two Ser/Thr kinases (PKA and p38MAPK). Residues of the DFG motif are boxed in red.

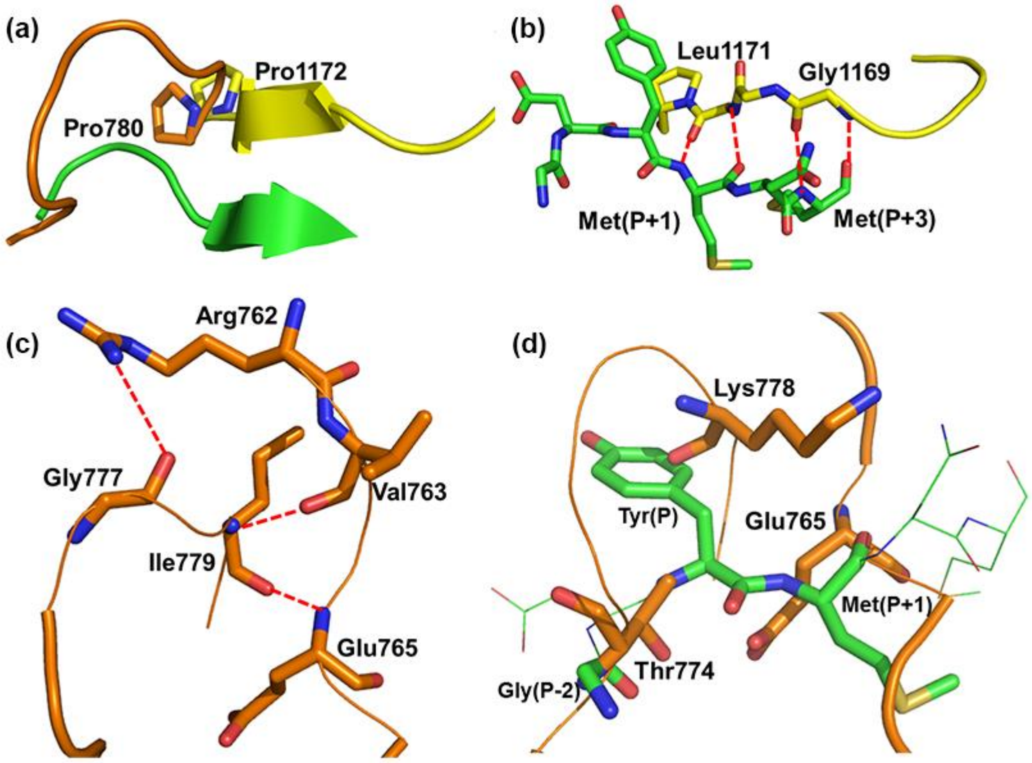

Figure S4. Conformation of the C-terminal part of the activation segment in the autoinhibited EphA2 structure and comparison with the ternary IRK3P structure (PDB 1IR3). a) The autoinhibited EphA2 structure is superposed with the ternary IRK3P structure, while most of the structures overlap well, the activation segment are in much different positions. The backbone of the activation segment of EphA2 is colored in orange, the IRK3P is colored yellow and the substrate peptide is shown in green. The PTK-invariant Pro residues in the two kinases are labeled. b) Selected hydrogen-bonding interactions for substrate accommodation in the IRK3P are shown as red dashed lines. Backbone hydrogen-bonding is observed between Met (P+1 residue) and Leu1171, and between Met (P+3 residue) and Gly1169. c) Selected hydrogen-bonding interactions for C-terminal residues of EphA2 activation segment are shown as red dashed lines. Backbone hydrogen-bond interactions are formed between Ile779 (corresponding to Leu1171 in IRK3P) and both Val763 and Glu765 from the activation loop, while Gly777 (corresponding to Gly1169 in IRK3P) forms a hydrogen-bonding interaction with Arg762. d) View of spatial clashes between the superimposed substrate and the activation segment of EphA2, especially between Lys778 and the superimposed P-Tyr (P residue), Thr774 and the Gly (P-2 residue), and Glu765 and the Met (P+1 residue).

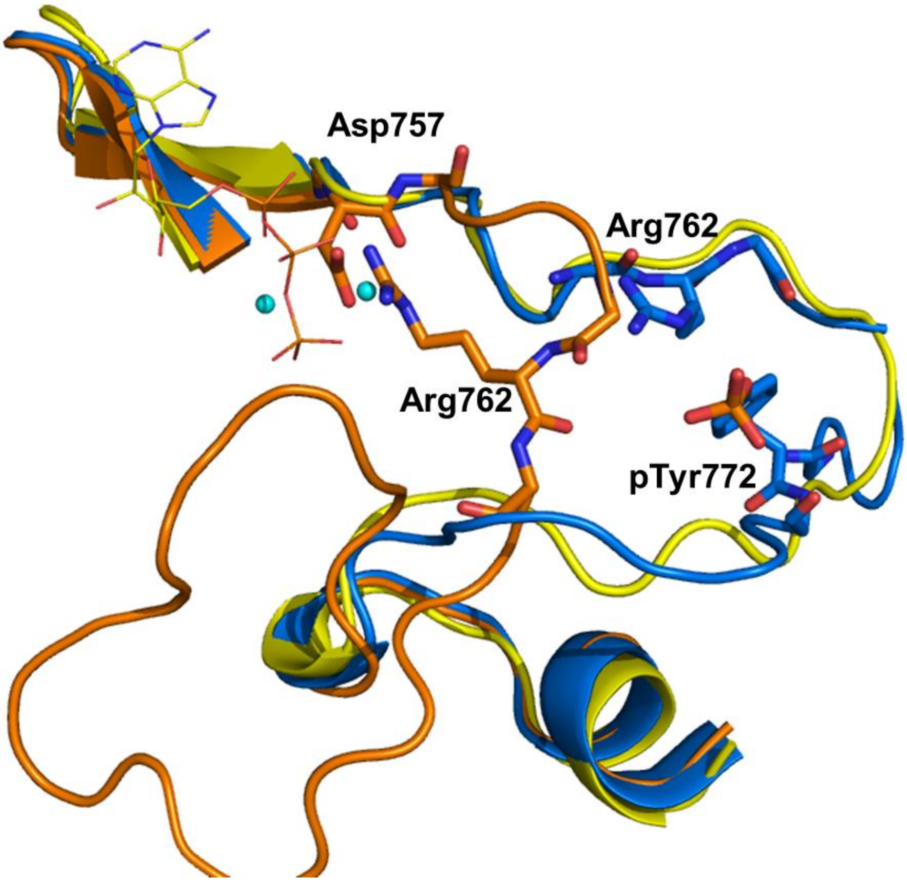

Figure S5. Alignment of the activation segment from the autoinhibited EphA2, the active EphA2, and the PKA. The backbones of the two EphA2 structures are colored as in Figure 3. The backbone of the PKA is yellow, and the Mg^{2+} ions are cyan.

Table S2 Effects of magnesium concentration on the relative velocities for wild-type and variant of EphA2, PKA and p38MAPK[a]

$[Mg^{2+}_{free}]$ (mM)	EphA2			PKA			p38MAPK		
	wild-type	Arg762Ala	p^{b}	wild-type	Lys189Ala	p^{b}	wild-type	Arg173Ala	p^{b}
0	0	0	-	0	0	-	0	0	-
0.5	0.11 ± 0.03	0.23 ± 0.02	0.005	0.14 ± 0.01	0.19 ± 0.02	0.03	0.27 ± 0.03	0.30 ± 0.01	0.13
0.8	0.26 ± 0.02	0.33 ± 0.02	0.02	0.31 ± 0.01	0.37 ± 0.03	0.03	0.38 ± 0.03	0.54 ± 0.01	0.001
1	0.30 ± 0.03	0.41 ± 0.02	0.005	0.38 ± 0.03	0.65 ± 0.03	<0.001	0.53 ± 0.01	0.69 ± 0.01	<0.001
1.2	0.38 ± 0.01	0.63 ± 0.03	<0.001	0.54 ± 0.04	0.73 ± 0.02	0.001	0.64 ± 0.02	0.87 ± 0.04	<0.001
1.5	0.70 ± 0.02	0.80 ± 0.02	0.002	0.72 ± 0.01	0.89 ± 0.02	<0.001	0.81 ± 0.02	0.89 ± 0.01	0.001
2	1	1	-	1	1	-	1	1	-

[a] Data are expressed as relative velocities, mean ± SD, $n = 3$.

[b] *p* values for the wild-type versus the variant at different magnesium concentrations are calculated by Student's *t* test (independent, two-tailed).

Table S3 Effects of magnesium concentration on the velocities for variants of EphA2, PKA and p38MAPK[a]

$[Mg^{2+}_{free}]$ (mM)	EphA2 Tyr772Phe	EphA2 Arg762Ala/Tyr772Phe	p[b]	PKA Thr197Ala	PKA Lys189Ala/Thr197Ala	p[b]	$[Mg^{2+}_{free}]$ (mM)	p38MAPK Thr180Ala	p38MAPK Arg173Ala/Thr180Ala	p[b]
0	0	0	-	0	0	-	0	0	0	-
0.5	0.10 ± 0.12	0.03 ± 0.01	0.35	0.15 ± 0.04	0.14 ± 0.02	0. 61	0.5	0.11 ± 0.01	0.10 ± 0.01	0.09
1	0.08 ± 0.05	0.05 ± 0.02	0.50	0.15 ± 0.004	0.24 ± 0.06	0.05	0.7	0.16 ± 0.03	0.19 ± 0.01	0.19
2	0.09 ± 0.11	0.28 ± 0.02	0.04	0.33 ± 0.05	1.09 ± 0.12	<0.001	0.9	0.20 ± 0.02	0.33 ± 0.05	0.02
3	0.31 ± 0.03	0.80 ± 0.18	0.01	0.79 ± 0.05	1.94 ± 0.15	<0.001	1.1	0.26 ± 0.03	0.45 ± 0.01	<0.001
5	0.51 ± 0.19	1.30 ± 0.08	0.003	0.93 ± 0.07	2.38 ± 0.11	<0.001	1.3	0.29 ± 0.03	0.48 ± 0.02	<0.001
7	0.82 ± 0.05	1.45 ± 0.12	0.001	1.05 ± 0.07	2.29 ± 0.36	0.004	1.5	0.34 ± 0.04	0.57 ± 0.08	0.01
9	0.89 ± 0.01	1.70 ± 0.02	<0.001	1.08 ± 0.06	2.43 ± 0.06	<0.001	1.7	0.33 ± 0.02	0.60 ± 0.05	<0.001
10	1.00 ± 0.06	1.69 ± 0.01	<0.001	1.13 ± 0.04	2.48 ± 0.09	<0.001	2	0.33 ± 0.03	0.60 ± 0.06	0.002

[a] Data are expressed as velocities (μM/min), mean ± SD, $n = 3$.

[b] p values for the single mutant versus the double mutant at different magnesium concentrations are calculated by Student's t test (independent, two-tailed).

Table S4 Steady-State Kinetic Parameters for Mutants of EphA2, PKA and p38MAPK[a]

parameter	EphA2			PKA			p38MAPK		
	Tyr772Phe	Arg762Ala/ Tyr772Phe	p[b]	Thr197Ala	Lys189Ala/ Thr197Ala	p[b]	Thr180Ala	Arg173Ala/ Thr180Ala	p[b]
$K_{peptide}$ (mM)	1.89 ±0.33	2.14 ±0.35	0.32	0.43 ±0.03	0.39 ±0.02	0.24	0.18 ±0.08	0.17 ±0.1	0.68
K_{ATP} (mM)	0.14 ±0.01	0.15 ±0.01	0.05	1.58 ±0.31	1.14 ±0.08	0.16	0.29 ±0.06	0.35 ±0.05	0.24

[a] Data are expressed as $K_{peptide}$ or K_{ATP} (mM), mean ± SD, n = 3.

[b] p values for the single mutant versus the double mutant of $K_{peptide}$ or K_{ATP} are calculated by Student's t test (independent, two-tailed).
